# Optimization of Compound Ratio of Exogenous Xylanase and Debranching Enzymes Supplemented in Corn-Based Broiler Diets Using In Vitro Simulated Gastrointestinal Digestion and Response Surface Methodology

**DOI:** 10.3390/ani12192641

**Published:** 2022-10-01

**Authors:** Wei Wu, Huajin Zhou, Yanhong Chen, Chunyue Li, Yuming Guo, Jianmin Yuan

**Affiliations:** State Key Laboratory of Animal Nutrition, College of Animal Science and Technology, China Agricultural University, 2 Yuanmingyuan West Road, Haidian District, Beijing 100193, China

**Keywords:** in vitro simulated digestion, corn, xylanase, zymogram, response surface methodology

## Abstract

**Simple Summary:**

Arabinoxylan (AX), making up 50% or more of the total carbohydrates, is the primary antinutritional factor in corn and related by-products. It is particularly fortified with substituents, being more populated than in other cereal grains. These properties may be overcome by the action of xylanase. More importantly, the complete enzymatic degradation of AX demands the synergistic combinations with debranching enzymes due to its complicated and multi-branched structure. We predicted the optimal zymogram of exogenous xylanase, arabinofuranosidase, and feruloyl esterase supplemented in corn AX for efficient hydrolysis using the in vitro simulated gastrointestinal digestion model, thus highlighting the compound complementarity of debranching enzymes. It was a bold technical attempt to rapidly target, alleviate or eliminate antinutritional factors by multi-enzyme cocktails based on substrate specificity. This may provide guidance for the efficient promotion and application of related feed ingredients to reduce excessive costs in the broilers industry.

**Abstract:**

This experiment aimed to explore the zymogram of endo-xylanase (EX) and debranching enzymes (arabinofuranosidase [EA] and ferulic acid esterase [EF]) supplemented in the corn–soybean meal-based diet of broilers. An in vitro simulated gastrointestinal digestion model was adopted. According to single-factor, completely random design, the optimal supplemental levels of individual carbohydrase were determined by reducing sugars (RS) and in vitro dry matter digestibility (IVDMD). Response surface method (RSM) was used to predict the proper compound ratio of three carbohydrases. Results showed that shifts were different for feedstuffs such as corn–soybean meal–distillers dried grains with solubles, corn hull, and wheat bran, revealing that the net increase of RS or IVDMD distinctly dropped when degrading corn and related by-products by EX (*p* < 0.05). There was a significant quadratic relationship between the above response metrics and addition levels of each enzyme (*p* < 0.05). The determined dosage was 54 U/g EX, 5.0 U/g EA, and 0.4 U/g of EF, respectively. The optimistic zymogram of carbohydrases in corn basal substrates was judged by the IVDMD screening (R^2^ = 0.9089, *p* < 0.001). Conclusively, the in vitro assay and RSM were convenient and rapid methods for the optimization of xylan-degrading zymogram, and also testified asthenic hydrolysis of corn arabinoxylan by EX, thus highlighting the synergistic combinations with debranching enzymes.

## 1. Introduction

Arabinoxylan (AX) is a primary antinutritional factor commonly present in corn and its milling by-products, which negatively influences nutrient digestibility and intestinal health in poultry. Compared with wheat and other cereals, AX in corn is particularly resistant to enzymatic disassembly. Although there have been numerous studies revealing the enzymatic hydrolysis behavior of supplemental endo-xylanase (EX), such effectiveness for both in vitro and in vivo settings were varied [1,2,3,4]. In addition, solitary EX has been proven to marginally degrade corn AX [5]. The nature and quantity of substituted side chains along linear backbones are dominant characteristics of AX [6], and present a great challenge to degradation. Thus, a complete hydrolysis of AX could most likely be obtained through the synergistic combinations of complementary EX and debranching enzymes such as arabinofuranosidase (EA) and ferulic acid esterase (EF), in terms of the presence of complex multi-branched structure [5,7,8].

This compound combination of specific depolymerizing carbohydrases, also called zymogram, synergistically acts on AX, aiding in the release of monosaccharides and the improvement of the degradation rate [9,10]. Therefore, EA removes the arabinose residues in order to expose more cleavage sites and enhance EX accessibility [11,12], while EF cleaves ferulic acid ester bonds cross-linked to arabinose residues in order to liberate ferulic acid [13,14]. Accordingly, our previous work systematically demonstrated that water-extractable AX and water-unextractable AX were hydrolyzed more effectively in vitro by the special xylanase collaborations with EA and EF [10]. Scanning electron microscopy further revealed a notable degradation of the honeycomb surface of the cell walls when wheat bran was exposed to the specific zymogram, whereas EX alone showed minimal visual changes. This further confirmed that debranching enzymes appreciably increased EX access and hemicelluloses degradability by removing those specific attachments and linkages [5]. More importantly, complicated AX in corn and its by-products is heavily fortified with substituents [15], being more populated than in wheat and other cereal grains. In-depth information on the synergistic potential of xylanase in combinations with both EA and EF may improve the nutritive value of corn based diets. Moreover, carbohydrases classified with different glycoside hydrolase families display different mechanisms of action and substrate specificities [7,12]. It is, therefore, crucial for achieving efficient AX hydrolysis to consider the synergistic complementarity and compound ratios between xylanase and debranching enzymes used to formulate enzyme cocktails.

In vitro assessments are simple to operate, less expensive, rapid, and not limited to use of animals compared to the in vivo experiments. It is an invaluable strategy to mimic in vivo conditions using the in vitro techniques, such as artificial gut models. The simulated models, including both the gastric and intestinal phases, have been applied to poultry research over the last few decades to predict the digestibility of nutrients, the efficacy of feed enzymes and additives, and fermentation concepts [16,17]. In order to exert the importance of these tools in poultry nutrition studies, nutritionists have developed and exploited several mature simulated digestion procedures for domestic birds [18,19]. Given that new enzyme preparations need to be screened and tested in large numbers of animals to determine their efficacy before they can be released into the commercial poultry market, the aforementioned model methods can potentially appraise enzyme dose rates and enzymatic hydrolysis effects before use in live chickens. In vitro assays determining the enzyme-substrate specificity are ordinarily judged by reducing sugars (RS) and in vitro dry matter digestibility (IVDMD) indicators. Moreover, Morgan et al. [20] concluded that the simulated gut model successfully predicted the influence of xylanase supplementation on xylooligosaccharide (XOS) production in the gastric and small intestine phase of broiler chickens, indicating that in vitro experiments used to evaluate enzyme effects may be correlated with in vivo observations. However, there is limited information on the synergistic effects of EX and debranching enzymes for corn AX considering diverse combinations or even multiple doses, and judgments associated with the enzymatic hydrolysis effect based on different response index values are particularly scarce.

Collectively, this study aimed to optimize the zymogram of three exogenous carbohydrases, including EX, EA, and EF, for the optimistic AX degradation in a corn–soybean meal basal diet of broilers, using the in vitro simulated gastrointestinal digestion method. These results may provide new approaches to screen the specific xylan-degrading enzymes for corn and its by-products, which could improve the degradation of corn AX for broilers via the novel complex feeding enzymes.

## 2. Materials and Methods

### 2.1. Feed Ingredients and Enzyme Preparations

A total of five feed ingredients were finely ground and passed through a 0.45 mm sieve, detected dry matter basis (DM, %) before enzymatic analysis. These included corn, soybean meal, distillers dried grains with solubles (DDGS), corn hull (CH), and wheat bran (WB). The exogenous carbohydrases used in the present study were manufactured via microbial engineering. We used *Aspergillus niger* derived xylanase (EX1), EA, and EF (90,000, 10,000, and 1000 U/g, respectively: Bestzyme Bio-products, Co., Ltd., Jinan, China). The other main xylanase (EX2; 200,000 U/g), produced from *Trichoderma* by Asiapac Co., Ltd. (Dongguan, China), was chosen for the subsequent evaluation of enzymatic hydrolysis.

### 2.2. The In Vitro Simulated Gastrointestinal Digestion (IVSGD) Model

All protocols were carried out in triplicate according to the 2-step assay suggested by Clunies and Leeson [21] with several modifications referring to the novel bionic digestion procedure [22]. Briefly, the gastrointestinal simulation buffer was prepared as follows to match in situ ionic concentrations of fluid from broiler chickens. pH was adjusted with HCl or NaOH solution. In order to mimic the gastric phase, 5.18 g of sodium chloride, 0.50 g of potassium chloride, and 15.60 g of sodium dihydrogen dihydrate were weighed and dissolved in distilled water to 1000 mL (pH 2.80 at 41 °C, refrigerated at 4 °C). In order to mimic the small intestine phase, 13.68 g of anhydrous disodium hydrogen phosphate and 50.44 g of sodium dihydrogen phosphate dihydrate were dissolved in distilled water to adjust to 1000 mL (pH 6.91 at 41 °C; room temperature stored).

Single or mixed ingredients substrates of accurate grams were weighed in 50 mL sterile enzyme-free Corning centrifuge tubes. Then, 8 mL of freshly prepared pepsin solution (0.2735 g pepsin slowly dissolved in 100 mL gastric buffer; ≥250 U/mg solid, Sigma P7000, pepsin from porcine gastric mucosa; St. Louis, MO, USA) and 1 mL of chloramphenicol solution (sterilization in system) were added. While shaking, 1 mL of appropriately diluted exogenous enzyme was quickly added, and the mixture was reacted in a water bath at 41 °C lasting for 4 h. During the second reaction stage, 6 mL of intestinal buffer and 1.6 mL of freshly prepared pancreatin solution (1.8660 g trypsin dissolved in 100 mL distilled water; Amresco 0458) were added. The test tubes were incubated in the same shaking bath at 41 °C for 15 h. All samples were placed in an ice bath to stop enzyme action. The supernatant was stored at 4 °C after centrifugation (5000× *g* for 10 min). The undigested residue was washed with distilled water three times, and then dried at 65 °C overnight and incubated at 105 °C for 4 h before analysis.

### 2.3. In Vitro Simulated Digestion of Different Feed Types Supplemented with Exogenous Xylanase

Using the single-factor, completely random design, two main xylanases (EX1/EX2) were employed separately in the main substrates of corn–soybean meal-DDGS, sole CH, or WB, each corresponding to 7 or 8 additional levels (Table 1). The blank control (CON) was treated with an equal volume of distilled water. The IVSGD model was applied to probe the enzymatic effects of the core xylanase on different substrate types.

**Table 1:** Different levels of core endo-xylanase added to multiple substrates.

Substrates	CON ^1^	Supplemental Levels of Enzyme, U/g						
		*Aspergillus niger* derived xylanase (EX1)						
Corn-DDGS	0	9	18	36	54	72	90	
Corn hull (CH)	0	9	18	36	45	54	72	90
Wheat bran (WB)	0	3	6	9	12	15	18	
		*Trichoderma* derived xylanase (EX2)						
Corn-DDGS	0	10	20	40	50	60	80	100
Corn hull (CH)	0	10	20	40	50	60	80	100
Wheat bran (WB)	0	5	10	20	30	40	50	60

^1^ CON: the blank control was treated with an equal volume of distilled water instead of exogenous enzymes. The same as follow in Table 2.

**Table 2:** Supplemental levels of single-factor experimental parameters (xylan-degrading enzymes) added to corn arabinoxylan ^1^.

Exogenous Enzymes ^2^	CON	Supplemental Levels, U/g					
EX	0	9	18	36	54	72	90
EA	0	2.0	3.0	4.0	5.0	6.0	7.0
EF	0	0.2	0.4	0.5	0.6	0.8	1.0

^1^ The substrates (dry matter basis) were composed of corn (70% *w*/*w*), soybean meal (25% *w*/*w*), and DDGS (5% *w*/*w*). ^2^ The EX (EX1), EA, and EF were produced from *Aspergillus niger*.

### 2.4. In Vitro Enzymatic Hydrolysis Assessment of Corn AX by Specific Xylan-Degrading Enzymes Combinations

The tested exogenous carbohydrases, including EX1, EA, and EF, were individually arranged into six supplemental levels in the complex substrates of corn and its by-products (Table 2), and the in vitro simulated digestion experiments were carried out for optimal potential of different enzyme supplementations. The substrates were consistently composed of corn (70% *w*/*w*), soybean meal (25% *w*/*w*), and DDGS (5% *w*/*w*). Therefore, response surface methodology with a five-level, three-variable Box–Behnken design (BBD) was employed and established by the Design Expert program (Version 8.0.6), requiring 23 different test groups for optimization of the specific arabinoxylan-degrading zymogram. Specifically, the above three carbohydrases were the three experimental factors, each factor corresponding to five levels, which were encoded by −1.682, −1, 0, 1, and 1.682 (Table 3). All test groups were performed according to the IVSGD method (Table 4). The RS (y_1_) and IVDMD (y_2_) were chosen as the response variables of the BBD experiments.

### 2.5. In Vitro Measurements and Calculation of Response Metrics

Based on the IVSGD method, the RS release of the digestion supernatant was determined using 3,5-dinitrosalicylic acid (DNS) [23]. IVDMD was analyzed by the weight loss between the DM of ingredients and undegraded samples. They were calculated by the following formulas:

RS (mg/g) = Y × XS_(n)_ × V/G_(DM)_ (1)

where Y is the DNS standard curve calculated value; XS_(n)_ is the dilution factor of supernatant samples during measurement; V represents the total volume of the solution reaction system (17.6 mL); and G_(DM)_ is the weight of substrates on DM basis (g).

IVDMD (%) = (IDM_i_ − DM_r_)/IDM_i_ × 100 (2)

where IDM_i_ is initial dry matter input (g) and DM_r_ is a dry matter of residue (g).

### 2.6. Statistical Analysis

The scatter diagrams presented the tendency of RS or IVDMD (mean ± standard error [SD]) with increasing additive levels of sole enzymes, and the linear or quadratic nature was performed by polynomial regression analysis (Version 21.0 for Windows, SPSS, Inc., Chicago, IL, USA). The fitting degree was expressed by coefficient R^2^ and *p*-value. A probability of *p* < 0.05 was considered statistically significant, and 0.05 < *p* < 0.10 was defined as a tendency towards significance. Considering the response surface method, the experimental design, model calculation, and graph drawing were performed by Design Expert Software (Version 8.0.6, Stat-Ease Inc., Minneapolis, MN, USA) [24].

## 3. Results

### 3.1. The Main Xylanases Displayed Low Degrees of Enzymatic Hydrolysis of Corn AX

**Figure 1:**
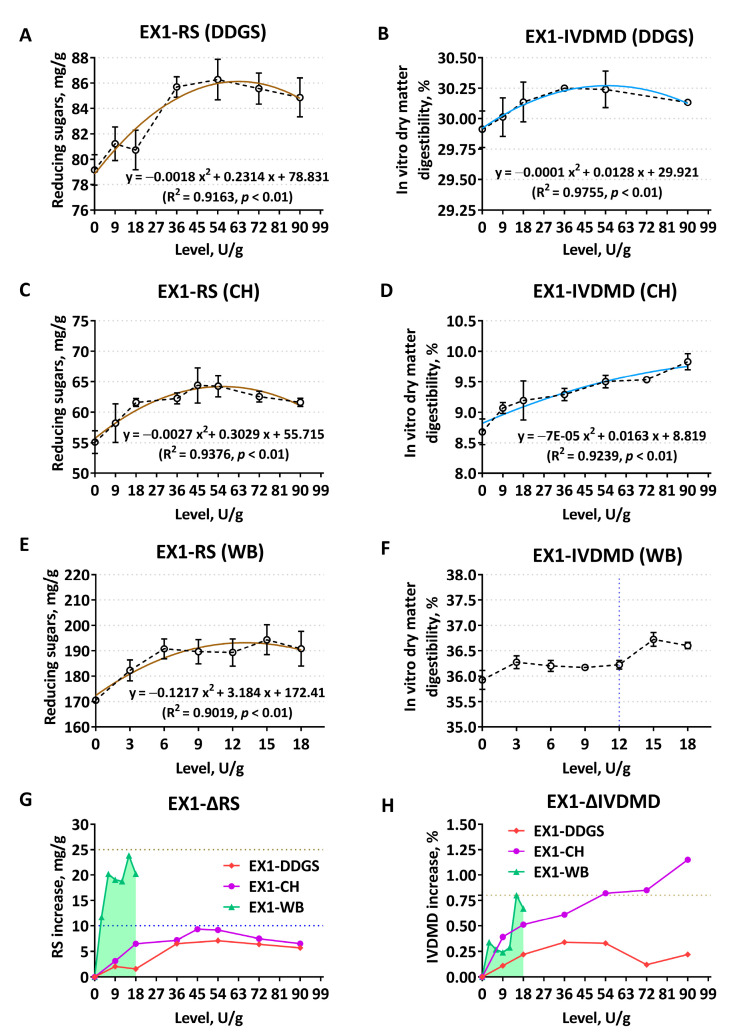
Comparison of enzymatic hydrolysis effects of *Aspergillus niger* derived xylanase EX1 on different feed ingredients, determined by the release of reducing sugars (RS) and the improvement of in vitro dry matter digestibility (IVDMD). (**A**,**B**) Corn–soybean meal basal substrates mixed with 5% DDGS; (**C**,**D**) unitary corn hull (CH) substrate; (**E**,**F**) unitary wheat bran (WB) substrate; (**G**,**H**) comparison of the net increase in RS release (ΔRS, mg/g) and dry matter digestibility (ΔIVDMD, %), respectively. Polylines directly explain the actual variability of each indicator. The dark brown curves in scatter plots display the RS trend, while the light blue represents the fit of IVDMD. The data are expressed as the means ± SD.

Based on the corn–soybean meal–DDGS composite substrates, with the increase of the supplement levels of EX1, both RS and IVDMD showed extremely significant quadratic curve changes (*p* < 0.01, R^2^ > 0.9) at the level of 54 U/g to achieve the highest enzymatic hydrolysis extent (Figure 1A,B). RS also showed a quadratic curve change (*p* < 0.01, R^2^ = 0.9376) when EX1 enzymatically hydrolyzed CH (Figure 1C). The degradation effect was higher at the addition level of 45 U/g and 54 U/g, while the increase in IVDMD was most typical at the highest level of 90 U/g (Figure 1D). The change trend of RS on WB was similar to the former two substrates (Figure 1E). The release amount was at its highest at an addition level of 15 U/g (*p* < 0.01, R^2^ = 0.9019), and the associated IVDMD reached its peak value (Figure 1F). When the equal supplement level of EX1 acted on CH substrate, the net increase of RS and IVDMD were higher than the corresponding changes of corn-DDGS, but the ΔRS did not exceed 10 mg/g, and the ΔIVDMD did not exceed 1.25% (Figure 1G,H). The degradation effect of the WB substrate by EX1 was obviously better than that of corn and its by-products (Figure 1G,H).

**Figure 2:**
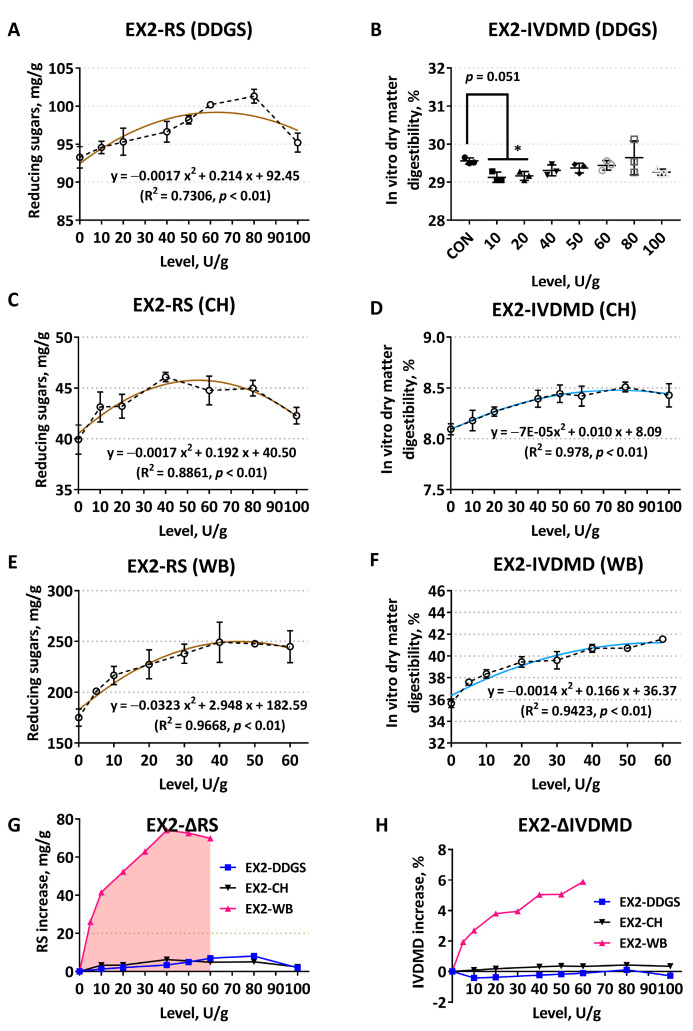
Comparison of enzymatic hydrolysis effects of *Trichoderma* derived xylanase EX2 on different feed ingredients. It was determined by the release of reducing sugars (RS) and the improvement of in vitro dry matter digestibility (IVDMD). (**A**,**B**) Corn–soybean meal basal substrates mixed with 5% DDGS; (**C**,**D**) unitary corn hull (CH) substrate; (**E**,**F**) unitary wheat bran (WB) substrate; (**G**,**H**) comparison of the net increase in RS release (ΔRS, mg/g) and dry matter digestibility (ΔIVDMD, %), respectively. Polylines directly explain the actual variability of each indicator. The dark brown curves in the scatter plot display the RS trend, while the light blue represents the fit of IVDMD. The data are expressed as the means ± SD. The asterisk (*) in this Figure 2B indicates that the corresponding response index values of the enzyme treatment groups are significantly different from the blank control (CON).

When EX2 enzymatically hydrolyzed corn-DDGS, RS enhanced gradually with the increase of supplementation levels, and the added amount of 80 U/g was more suitable (*p* < 0.05) (Figure 2A). However, there were no significant linear or quadratic changes in IVDMD under this condition, and the degradation degree even decreased compared with the CON (Figure 2B). Response metrics showed extremely significant quadratic curve changes when CH was used as the substrate (Figure 2C,D). RS reached a peak value with the exogenous addition of 40 U/g, and the addition of 80U/g had the highest in vitro degradation rate of the substrate. When enzymatically hydrolyzing WB, RS and IVDMD also showed notable quadratic curve changes (*p* < 0.01, R^2^ > 0.9), and the enzymatic hydrolysis effects were significantly improved at 40–60 U/g (Figure 2E,F). As shown in Figure 2G, when the same addition level of EX2 acted on corn DDGS and CH substrates, respectively, the corresponding ΔRS were relatively close and low. The ΔRS and ΔIVDMD of the WB substrate exogenously supplemented with EX2 (5 U/g) were significantly higher than the corresponding values of the corn DDGS and CH substrates (*p* < 0.05) (Figure 2G,H).

### 3.2. Optimization of Individually Exogenous Xylanase or Debranching Enzymes in Corn–Soybean Meal–DDGS Composite Substrates

**Figure 3:**
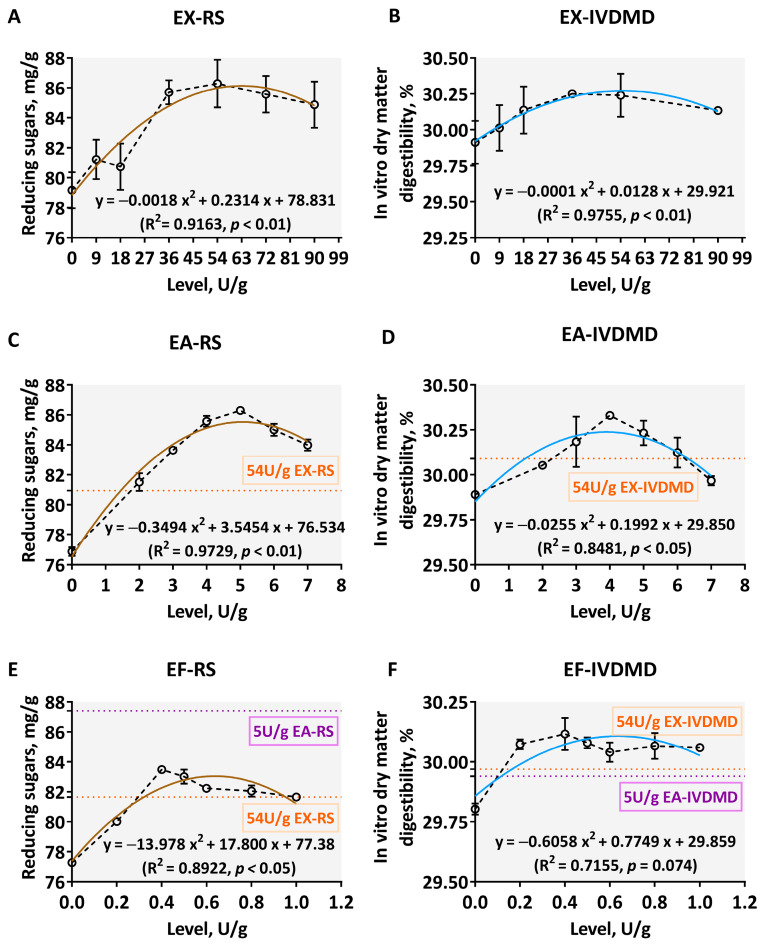
Disassembly degrees of corn AX by different levels of xylan-degrading enzymes. The core EX (**A**,**B**) and two debranching enzymes ((**C**,**D**): EA; (**E**,**F**): EF were produced from *Aspergillus niger*. The dark brown curves in scatter plots (**A**,**C**,**E**) display the reducing sugars (RS) trend, while the light blue (**B**,**D**,**F**) represents the fit of in vitro dry matter digestibility (IVDMD). The orange and purple dotted lines show RS or IVDMD indicators corresponding to the hydrolysis of 54 U/g EX and 5.0 U/g EA for this composite substrate, respectively. The data are expressed as the means ± SD.

The RS and IVDMD of the corn–soybean meal–DDGS composite substrates supplemented with tested exogenous carbohydrase individual were shown in Figure 3. Significant quadratic relationships were found between the supplemental levels of exogenous carbohydrase individuals and RS as well as IVDMD (*p* < 0.05, R^2^ > 0.8). As the supplement levels of xylanase or debranching enzymes increased within the tested range, the two types of response indicators first increased, then decreased. When the supplement level of EX reached 54 U/g, the improved RS and IVDMD reached their maximums of 86.28 mg/g and 30.24%, respectively (Figure 3A,B). When the supplement levels of EA and EF reached 5.0 U/g and 0.4 U/g, the RS values reached their highest levels of 86.29 mg/g and 83.49 mg/g, respectively (Figure 3C,E). Moreover, the associated improved IVDMD reached its highest levels of 30.33% and 30.12%, respectively (Figure 3D,F). In addition, compared with the effect of the appropriate level (54 U/g) of EX, when the addition level of EA reached a range from 2.0 to 7.0 U/g, the RS was higher than the corresponding level of EX (average 80.95 mg/g) (Figure 3C). However, compared to the effect of EA at the appropriate level (5.0 U/g) obtained from the above investigation, the amount of RS produced by EF was far lower than its corresponding levels (average 87.40 mg/g) (Figure 3E).

### 3.3. Response Surface Analysis of RS and IVDMD of Corn–Soybean Meal–DDGS Composite Substrates Supplemented with the Xylan-Degrading Zymogram

The Box–Behnken design of three exogenous carbohydrases and the predicted RS and IVDMD for 23 experiments were shown in Table 4. Of the 23 tests in Table 4, the test number 20 (54 U/g EX, 5.0 U/g EA, 0.4 U/g EF) and the test number 1 (63 U/g EX, 6.0 U/g EA, 0.5 U/g EF) produced the maximum amount of RS (75.89 mg/g) and IVDMD (36.60%), respectively. Table 5 presented the results of the ANOVA of the quadratic polynomial model for optimization of the exogenous carbohydrases zymogram in order to achieve optimal IVDMD in corn–soybean meal–DDGS composite substrates. The *F*-value of the model (*F*_2_) was 14.41, and the *p*-value was less than 0.01 (Table 5), indicating that the quadratic model was reliable only for each X_i_ factor. The lack of *F*-value (*F*_1_) was 1.09, and the *p*-value was 0.41 (*p* > 0.05), representing that the model misfit was insignificant. However, EF accounted for more influence than EA and EX did on IVDMD (Figure 3F) by comparison of mean square values for each xylan-degrading enzyme in Table 5. The interaction of X_1_X_2_ was self-evident at α = 0.10 (*p* < 0.05), indicating that IVDMD manifested as an exaltation by increasing the levels of EX and EA in certain ranges. The regression equation of IVDMD (y_2_) and xylan-degrading enzymes (X_i_) was as follows:

y_2_ = 32.773 + 0.248 X_1_ + 0.253 X_2_ + 0.267 X_3_ + 0.404 X_1_^2^ + 0.622 X_2_^2^
 + 0.944 X_3_^2^ + 0.410 X_1_X_2_ + 0.113 X_1_X_3_ + 0.220 X_2_X_3_ (R^2^ = 0.9089). (3)

Nevertheless, it was preliminarily considered that the regression model of RS (y_1_) was unreasonable according to the *F*-test (Table 6). The mathematical model for prediction was insignificant (*p* = 0.0620), and the Lack of Fit of the model was significant (*p* = 0.0015).

**Figure 4:**
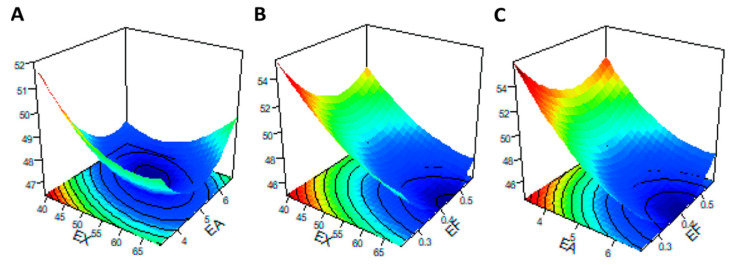
Response surface and contour plots of the interaction effects between enzyme addition levels for factors [(**A**): EX/EA; (**B**) EX/EF; (**C**) EA/EF] on the in vitro dry matter digestibility (y_2_). The abscissa axis at the bottom of the figure represents the two enzyme factors, respectively, and the vertical axis upward represents the corresponding response index (y). The associated contour plots are composed of the densely arranged circles on the underside.

**Figure 5:**
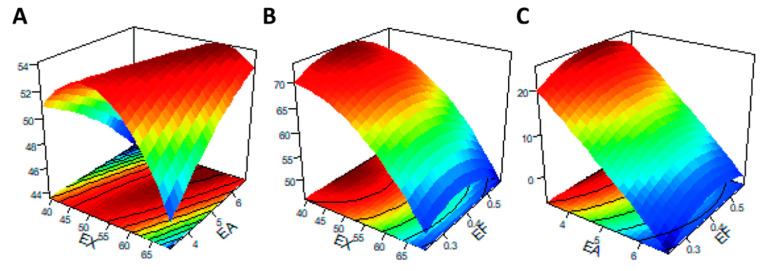
Response surface and contour plots of the interaction effects between enzyme addition levels for factors [(**A**): EX/EA; (**B**) EX/EF; (**C**) EA/EF] on the reducing sugars (y_1_). The abscissa axis at the bottom of the figure represents the two enzyme factors, respectively, and the vertical axis upward represents the corresponding response index (y). The associated contour plots were composed of the densely arranged circles on the underside.

Three-dimensional (3D) images of the response surface were generated to demonstrate significant interactions between the tested carbohydrases and the response metrics. When any one of the three variables was selected as zero level, the effect of simultaneous changes in the other two variables on IVDMD is shown in Figure 4. When the supplementary level of EF was fixed at 0.4 U/g, as obtained from the slope of the response surface (Figure 4A), EX and EA had significant interaction effects on IVDMD, which was consistent with the mentioned results of ANOVA (Table 5). In addition, when the addition level of EX was less than 50 U/g, and the corresponding level of EA was less than 5 U/g, the contour lines were denser (Figure 5A), indicating that the interaction between EX and EA had a significant impact on RS within this range.

## 4. Discussion

Xylanase is thought to be a candidate to replace antibiotic growth promoters, and degrades AX in commercial corn or wheat basal diets for broilers [10,25,26]. Thus, an effective approach by xylanase supplementation could alleviate or even solve the antinutritional problems [1,2]. Differences in AX structure among cereals and their by-products are facilitated by many factors, such as cereal types, degree and pattern of substitution along the xylan backbone, phenol content, and cross linkages [5]. In order to elucidate the enzymatic hydrolysis effects of individual xylanase on different AX types of feed ingredients, RS and IVDMD were selected as the major measurements through the current in vitro evaluation. Regardless of substrate basis, RS showed significant quadratic curve changes with additional levels of EX1, which was similar to the related tendency of EX2. However, the IVDMD values of EX1 acting on different types of feedstuffs did not show more consistent regularity, and the same was true for EX2. It suggests that the overall effects of in vitro judgment based on different indicators or substrates type are difficult to keep consistent. More interestingly, net increases in ΔRS and ΔIVDMD of CH were slightly higher than those of corn DDGS substrates degraded by the equivalent concentration of EX1, which were relatively minimal compared to the CON group. On the contrary, the degradation of corn and its by-products was exceeded by the corresponding changes in WB, which was explained by higher extents at 6–15 U/g EX1. Compared with bran, the weaker enzymatic hydrolysis of EX2 on corn and its by-products was similarly revealed. This may be related to the abundant proportion of NSP and AX in wheat grains [27]. The difference in the hydrolysis effect of an individual xylanase on various substrate types may depend on insoluble AX content in common cereals. Furthermore, there is clear evidence of variation in spatial distribution among cereal species. AX in corn is particularly resistant to enzymatic hydrolysis, owing to its unique chemical composition and heavily branched substituents [28,29]. Constructed as such, the ability of commonly used feed-grade EX to degrade corn AX is greatly impaired [29,30,31,32]. Given the outcomes of two microbial sources of xylanases (EX1/EX2) on corn substrates, EX1 was considered for continuation of the follow-up test.

Debranching enzymes, such as EA and EF, were co-opted under the premise of xylanase, aiming to further upgrade the hydrolysis of AX in corn and byproducts, as well as to obtain the suitable specific AX degrading zymogram by the IVSGD and response surface methodology. Varying amounts of enzyme preparations used have different effects. There is no generally accepted standard for measuring the levels of enzyme supplementation. This is because the amount of enzyme added needs to be determined comprehensively, considering enzyme activity, substrates, and environmental conditions [2]. The activity of enzymes is affected by many factors, such as enzyme source, concentration, substrate, temperature, and pH. For the single-factor experiments in this study, it was found that effects of EX, EA, and EF on the RS release or IVDMD were significant with respective increased levels of addition in corn AX, accompanied by quadratic curve trends. This phenomenon was consistent with strong biological statistical significance. Based on the data regarding quadratic regression equations, the optimal supplement levels of each exogenous carbohydrase for the response metrics were acquired, and it was found that 54 U/g EX, 5.0 U/g EA, and 0.4 U/g EF could improve the RS and IVDMD of corn–soybean meal–DDGS composite substrates to the maximum. Therefore, the enzyme supplementations EX (54 U/g), EA (5.0 U/g), and EF (0.4 U/g) were selected for the response surface experiment by BBD design. Notably, the hydrolysis of a unitary debranching enzyme was more vigorous than that of xylanase within certain dose levels in our study. Concern has also previously been raised that debranching enzymes worked in collaboration with core enzymes to increase enzymatic accessibility and to improve the efficiency of carbohydrases such as EX [5].

In the future, zymogram mixtures are projected not to differ so much in the core xylanase, but rather, in conjunction with debranching enzymes to attack side chains or phenolic linkages [33,34]. In mixtures with comparable cellulase or xylanase, for example, the debranching enzymes markedly increased in vitro enzymatic hydrolysis over that of the primary enzymes in sorghum bagasse or corn stalk, as well as corn AX [35]. However, understanding the interactions between individual enzymes in order to explore the synergistic effects and avoid antagonism is key, and cannot be ignored when using compound enzyme preparations. In vitro studies have disclosed synergism between EA and EX for AX disintegration in wheat as well as corn [36,37]. This collaboration between xylanase and debranching enzymes occurs with both soluble and insoluble portions of AX, suggesting this synergism could exist in either corn- or wheat-based diets [37]. In the present study, BBD for response surface methodology was employed to predict the optimistic combination of EX, EA, and EF for AX digestion in corn and by-product substrates. It was shown that EF accounted for more influence than EA and EX on IVDMD by comparison of mean square values based on analysis of variance of a regression model. The interaction of variables X_1_X_2_ was self-evident at α = 0.10, indicating that IVDMD manifested as an exaltation by increasing the levels of EX and EA in certain ranges. Response surface 3D images and contour maps of X_1_X_2_, X_1_X_3_, and X_2_X_3_ were selected in order to visually represent the interaction of these factors on RS or IVDMD indicators in this study. The steeper the slope of the response surface, the more sensitive it is to the changes of other factors, and the greater the impact on the Y index [24]. Contour lines also intuitively reflected the significance of the interaction between two factors. An ellipse indicated the significant effect between the two factors, while a close circle indicated an insignificant effect. Regarding IVDMD values, only interactions between X_1_ (EX) and X_2_ (EA) showed significant effects, which were consistently demonstrated in the results of ANOVA. Moreover, effects of the interaction between tested exogenous EX and EA on RS were significant. However, the actual efficacy of the combination of experimented exogenous carbohydrases on the AX digestion of broiler diets still requires further study before popularizing in the feed formula. The above research and the data of this experiment show that the appropriate ratios of exogenous enzymes with different addition amounts and types were vital for improvement of the digestion of nutrients in the feed, so as to highlight the complementary effects.

## 5. Conclusions

In this study, by using in vitro simulated gastrointestinal digestion and response surface methodology, we predicted the optimal zymogram of EX and debranching enzymes supplemented in corn AX, which were 54 U/g *Aspergillus niger* derived EX, 5.0 U/g EA, and 0.4 U/g EF, in order to increase reducing sugar release and dry matter digestibility. It was inductively recommended to judge the combinations of corn-type AX-degrading enzymes, referring to a reasonable and reliable prediction model.

## Figures and Tables

**Table 3:** Independent variables (X_i_) and their ranges used for the zymogram optimization in the Box–Behnken design.

Carbohydrases Factors (X_i_)	Interval	Coded and Actual Levels, U/g (*p* = 3, γ = 1.682) ^1^				
		−γ	−1	0	+1	+γ
X_1_ EX	9	39	45	54	63	69
X_2_ EA	1.0	3.3	4.0	5.0	6.0	6.7
X_3_ EF	0.1	0.23	0.30	0.40	0.50	0.57

^1^ Both the asterisk arm value (γ) and the coded level values are defined by the Design Expert Software by means of BBD design. Ternary factors (*p*) include EX, EA, and EF.

**Table 4:** Box–Behnken design of exogenous carbohydrases with coded values and predicted response index values (y).

Tests	Supplemental Levels, U/g			y_1_	y_2_
	X_1_	X_2_	X_3_	Reducing Sugars, mg/g	In Vitro Dry Matter Digestibility, %
1	63.00	6.00	0.50	67.83	36.60
2	63.00	6.00	0.30	72.69	35.40
3	63.00	4.00	0.50	67.54	34.45
4	63.00	4.00	0.30	69.24	34.46
5	45.00	6.00	0.50	68.32	34.77
6	45.00	6.00	0.30	69.21	34.35
7	45.00	4.00	0.50	70.06	34.59
8	45.00	4.00	0.30	74.04	34.72
9	39.00	5.00	0.40	70.72	33.40
10	69.00	5.00	0.40	71.37	33.94
11	54.00	3.30	0.40	74.32	34.12
12	54.00	6.70	0.40	75.72	34.45
13	54.00	5.00	0.23	70.84	34.55
14	54.00	5.00	0.57	72.54	35.84
15	54.00	5.00	0.40	73.97	33.04
16	54.00	5.00	0.40	75.79	33.46
17	54.00	5.00	0.40	73.85	32.34
18	54.00	5.00	0.40	75.44	32.27
19	54.00	5.00	0.40	73.70	32.69
20	54.00	5.00	0.40	75.89	33.31
21	54.00	5.00	0.40	73.18	32.22
22	54.00	5.00	0.40	73.88	32.75
23	54.00	5.00	0.40	73.36	32.96

**Table 5:** Analysis of variance of a regression model based on in vitro dry matter digestibility (y_2_).

Variables	Sum of Squares	Degrees of Freedom	Mean Square ^1^	Partial Correlation	*F*-Value ^2^	*p*-Value
X_1_	0.8406	1	0.8406	0.4857	4.0143	0.0664
X_2_	0.8740	1	0.8740	0.4930	4.1741	0.0619
X_3_	0.9753	1	0.9753	0.5136	4.6578	0.0502
X_1_ ^2^	2.6006	1	2.6006	0.6990	12.4197	0.0037
X_2_ ^2^	6.1470	1	6.1470	0.8325	29.3565	0.0001
X_3_ ^2^	14.1505	1	14.1505	0.9158	67.5797	0.0001
X_1_X_2_	1.3448	1	1.3448	0.5750	6.4225	0.0249
X_1_X_3_	0.1012	1	0.1012	0.1894	0.4835	0.4991
X_2_X_3_	0.3872	1	0.3872	0.3529	1.8492	0.1970
Regression	27.1532	9	3.0170	*F_2_* = 14.40859		0.0002
Remainder	2.7221	13	0.2094			
Misfit	1.1025	5	0.2205	*F_1_* = 1.08921		0.4114
Error	1.6196	8	0.2024			
Sum	29.8752	22				

^1^ MS, mean square values. ^2^ A preliminary determination of the suitability of the selected regression model was made according to the misfit test of equation (*F_1_* = MS_misfit_/MS_error_). The significance test of the regression equation was measured by *F_2_*, which equated to the ratio of MS_regression_ to MS_remainder_. The significant difference between the regression model and the coefficient was evaluated by the *F* test, and *p* < 0.05 was considered significant.

**Table 6:** Analysis of variance of a regression model based on reducing sugars (y_1_).

Variables	Sum of Squares	Degrees of Freedom	Mean Square ^1^	Partial Correlation	*F*-Value ^2^	*p*-Value
X_1_	0.7670	1	0.7670	−0.1207	0.1922	0.6683
X_2_	0.0165	1	0.0165	−0.0178	0.0041	0.9497
X_3_	5.3781	1	5.3781	−0.3065	1.3478	0.2665
X_1_^2^	46.2768	1	46.2768	−0.6866	11.5971	0.0047
X_2_^2^	1.4423	1	1.4423	−0.1645	0.3614	0.5580
X_3_^2^	34.7362	1	34.7362	−0.6333	8.7050	0.0113
X_1_X_2_	13.2870	1	13.2870	0.4516	3.3298	0.0911
X_1_X_3_	0.3570	1	0.3570	−0.0827	0.0895	0.7696
X_2_X_3_	0.0006	1	0.0006	−0.0034	0.0002	0.9903
Regression	101.4984	9	11.2776	*F*_2_ = 2.82620		0.0620
Remainder	51.8748	13	3.9904			
Misfit	42.8556	5	8.5711	*F*_1_ = 7.60255		0.0015
Error	9.0192	8	1.1274			
Sum	153.3731	22				

^1^ MS, mean square values. ^2^ A preliminary determination of the suitability of the selected regression model was made according to the misfit test of equation (*F*_1_ = MS_misfit_/MS_error_). The significance test of the regression equation was measured by *F*_2_, which equated to the ratio of MS_regression_ to MS_remainder_. The significant difference between the regression model and the coefficient was evaluated by the *F* test, and *p* < 0.05 was considered significant.

## Data Availability

The minimal dataset that supports the central findings of this study is available on request from the corresponding author.

## Abbreviations

NSP: nonstarch polysaccharides; AX, arabinoxylan; DDGS, distillers dried grains with solubles; CH, corn hull; WB, wheat bran; EX, endo-xylanases; EA, arabinofuranosidase; EF, ferulic acid esterase; IVSGD, in vitro simulated gastrointestinal digestion; BBD, Box-Behnken design; RS, reducing sugars; IVDMD, in vitro dry matter digestibility; RSM, response surface method; XOS, xylooligosaccharides; SD, standard error.
